# Vitamin D metabolism-related single nucleotide polymorphisms in Chronic Obstructive Pulmonary Disease risk

**DOI:** 10.3389/fendo.2024.1445712

**Published:** 2024-11-08

**Authors:** Susana Rojo-Tolosa, Laura Elena Pineda-Lancheros, Andrea Fernández-Alonso, Noelia Márquez-Pete, Yasmin Cura, Cristina Membrive-Jiménez, Luciana Maria Iglecias-Marangoni, MCarmen Ramírez-Tortosa, José María Gálvez-Navas, Cristina Pérez-Ramírez, Concepción Morales-García, Alberto Jiménez-Morales

**Affiliations:** ^1^ Pneumology Service, University Hospital Virgen de las Nieves, Granada, Spain; ^2^ Facultad de Ciencias, Universidad Nacional de Colombia, Bogotá, Colombia; ^3^ Pharmacogenetics Unit, Pharmacy Service, University Hospital Virgen de las Nieves, Granada, Spain; ^4^ Federal University of Mato Grosso do Sul University Hospital, Campo Grande, Brazil; ^5^ Department of Biochemistry and Molecular Biology II, Faculty of Pharmacy, University of Granada, Granada, Spain; ^6^ Centro de Investigación Biomédica en Red de Epidemiología y Salud Pública (CIBERESP), Madrid, Spain; ^7^ Cancer Registry of Granada, Andalusian School of Public Health, Granada, Spain; ^8^ Instituto de Investigación Biosanitaria ibs.GRANADA, Granada, Spain

**Keywords:** chronic obstructive pulmonary disease, vitamin d, metabolism, single nucleotide polymorphisms, biomarkers, risk

## Abstract

**Introduction:**

Chronic obstructive pulmonary disease (COPD) is one of the world’s major public health problems. It is characterized by a major inflammatory response, where vitamin D, due to its role in regulating the immune system, and genetic variants involved in its metabolism may play an essential role. The aim of this study is to evaluate the association between 13 polymorphisms related to vitamin D metabolism and the COPD risk.

**Material and methods:**

A retrospective longitudinal study was designed in which 152 cases of COPD diagnosed at the University Hospital Virgen de las Nieves and 456 controls without the pathology, matched by age and sex, were included. The determination of the 13 polymorphisms was carried out using TaqMan™ probes.

**Results:**

Statistical analysis showed that the AA genotype and the A allele of the *CYP27B1* rs4646536 polymorphism may be associated with an increased risk of developing COPD according to genotypic models (OR = 2. 6; 95% CI = 1.38-5.22; p = 0.004), dominant (OR = 1.69; 95% CI = 1.15-2.5; p = 0.008), recessive (OR = 2.24; 95% CI = 1.22-4.41; p = 0.013) and additive (OR = 1.56; 95% CI = 1.18-2.08; p = 0.020) models. Likewise, the AA genotype and the A allele of the *CYP2R1* rs10741657 polymorphism were also associated with the risk of developing COPD according to the genotypic (OR = 1.9; 95% CI = 1.06-3.36; p = 0.028) and additive (OR = 1.37; 95% CI = 1.04-1.81; p = 0.027) models. Likewise, an association was found between GATG (p = 0.002; OR = 2.05; 95%CI = 1.32-3.20) and AGGT (p < 0.0001; OR = 2.1e46; 95%CI = 2.1e46-2.1e46) haplotypes and an increased risk of COPD.

**Conclusions:**

We can therefore conclude that those variants could be used in the early detection of the disease in the future.

## 1 Introduction

Chronic obstructive pulmonary disease (COPD) is a heterogeneous and complex disease with significant morbidity and mortality (1). It is a chronic respiratory disease characterized by persistent breathing difficulty, airflow limitation, and aberrant inflammation (2). Symptoms include shortness of breath, cough, sputum production, and wheezing (3). COPD is a major health problem worldwide due to its high prevalence of approximately 10% in the adult population, increased incidence, and high personal, social, and economic costs. It is considered one of the leading causes of premature death and the second most frequent respiratory disease in the world, it is estimated that COPD affected over 380 million people in 2022 (4).

COPD results from gene-environment interactions throughout the individual’s life that can alter the normal development/aging processes, leading to lung damage. The main environmental causes of COPD are smoking and exposure to harmful particles or gases from pollution. The most relevant COPD genetic risk factor identified to date is mutations in the *SERPINA1* gene, leading to α-1-antitrypsin deficiency. However, other genetic variants with a smaller individual effect size are also associated with reduced lung function and COPD risk. Evidence of an association between low concentrations of calcidiol (25(OH)D), the main circulating metabolite of vitamin D, and the severity of COPD patients has been reported (4, 5). Vitamin D is involved in various processes, such as immunity, inflammation, and lung biology. The presence of vitamin D receptors (VDR) and the enzyme responsible for its definitive activation in immune cells and the high prevalence of deficient levels of vitamin D in COPD patients suggest the involvement of vitamin D in the risk of this disease (6).

Vitamin D has two physiologically relevant and biologically inactive forms: D3 or cholecalciferol, of animal origin, which can be synthesized through exposure to UVB rays, and D2 or ergocalciferol, obtained from plant sources. Vitamin D metabolism has three main steps, all regulated by cytochrome P450 (CYP450) enzymes. Vitamin D3 produced in the dermis is transported through the blood, bound to the vitamin D binding protein (VDBP) encoded by the GC gene (7). The first hydroxylation, catalyzed by CYP2R1, occurs in the liver and transforms D3 into 25(OH)D. Subsequently, calcidiol is transported to the kidney, where it undergoes a second hydroxylation through CYP27B1 to form the active metabolite of vitamin D: 1,25(OH)2D3 or calcitriol (8, 9). Once calcitriol is produced, it binds to the VDBP protein and is transported to the target cells and tissues, where it binds to the VDR, forming a complex. It enters the nucleus and forms a heterodimer with the retinoid X receptor (RXR), which will regulate vitamin D-dependent genes (10). Finally, a hydroxylation regulated by CYP24A1 occurs, which converts the active form of vitamin D into calcitrioic acid, an inactive metabolite to be excreted (8).

The genes responsible for coding enzymes involved in vitamin D metabolism are very polymorphic (10–12). The presence of single nucleotide polymorphisms (SNPs) in the genes involved in vitamin D metabolism (*GC*, *CYP27B1*, *CYP24A1*, *CYP2R1*, and *VDR*) could crucially influence its activity and, therefore, they would represent a risk factor for developing COPD.

Considering the above, the aim of this study was to evaluate the effect of SNPs on genes involved in vitamin D metabolism with the risk of developing COPD in Caucasian patients from southern Spain, the first conducted in this population.

## 2 Materials and methods

A retrospective case-control study was conducted.

### 2.1 Ethical statements

This study was approved by the Ethical Committee of the Andalusian Health System and conducted in accordance with the Declaration of Helsinki (code: 0201-N-23). All subjects participating in the study signed a written informed consent for the collection of saliva or blood samples and their donation to the biobank. The samples were encoded and treated confidentially throughout the study.

### 2.2 Study subjects

This study included 152 patients with COPD and 456 controls of Caucasian origin from southern Spain; the case-control ratio was 1:3. The cases were diagnosed and recruited at the Virgen de las Nieves University Hospital, Granada (Spain), between 2017 and 2023. The cases met the following inclusion criteria to enter the study: Age ≥ 18 years and a diagnosis of COPD according to the guidelines of the Spanish COPD Guidelines (GesEPOC) (13). The specific diagnostic criteria established by GesEPOC are: 1) previous exposure to risk factors (e.g. smokers, former-smokers, inhalation of toxic agents); 2) respiratory symptoms (e.g. dyspnea or chronic cough); 3) obstruction in post-bronchodilator spirometry (FEV1/FVC < 0.7). The control group consisted of individuals over 18 years old, recruited at the same hospital, living in the same geographical area, and without a diagnosis of COPD, respiratory diseases, and others respiratory-related chronic diseases.

### 2.3 Sociodemographic and clinical variables

The sociodemographic and clinical data included sex, age, smoking habit, body mass index (BMI), alcohol habit, COPD phenotype, emphysema, chronic bronchitis, bronchial hyperreactivity, home oxygen (O_2_) therapy, respiratory insufficiency, dyspnea, exacerbation, O_2_ saturation, lung function, respiratory infection, pulmonary hypertension (PHT), osteoporosis, obstructive sleep apnea syndrome (OSA). Individuals were classified as non-smokers if they had never smoked or smoked < 100 cigarettes in their lifetime as former-smokers if they had smoked ≥ 100 cigarettes in their lifetime but stopped smoking at least 6 months ago. currently do not smoke; and as active smokers if they had smoked ≥ 100 cigarettes in their lifetime and were currently smoking (14). Individuals were classified according to their BMI following the criteria of the Spanish Society for the Study of Obesity: Insufficient weight (BMI < 18.5), healthy weight (18.5 < BMI < 24.9), overweight (25 < BMI < 29.9), and obesity (BMI > 30) (15). Individuals were classified based on the standard drink (SD) as a) non-drinkers if they were abstainers or did not consume alcohol regularly, b) active drinkers if their alcohol consumption was > 4 SD/day in men and > 2.5 SD/day in women, and c) former drinkers if their alcohol consumption was > 4 SD/day in men and > 2.5 SD/day in women, but they were not currently drinking (16). The disease phenotype was classified as exacerbator, non-exacerbator, and asthma-COPD overlap (ACO) (17). Hypertension, emphysema, chronic bronchitis, bronchial hyperreactivity, home O_2_ use, respiratory insufficiency, dyspnea, exacerbation, respiratory infection, HTP, osteoporosis, and OSA were evaluated as presence or absence (Yes/No) at the time prior to diagnosis. Saturation of O_2_ as %O_2_ in blood. The percentage of the maximum exhaled air volume during the first second of forced expiration (percentage of forced expiratory volume in 1 second (%FEV1)) was used to evaluate lung function. COPD severity was classified according to the Global Initiative for Chronic Obstructive Lung Disease (GOLD) standards, which uses FEV1 post-bronchodilator values and divides severity into 4 groups: 1) GOLD1. Mild FEV1 ≥ 80%; 2) GOLD2. Moderate 50% ≤ FEV1 < 80%; 3) GOLD3. Severe, 30% ≤ FEV1 < 50%; 4) GOLD4. Very severe, FEV1 < 30% (13).

All sociodemographic and clinical variables were collected through Diraya clinical software, and those mentioned in the cases were collected at the time of diagnosis.

### 2.4 Genetic variables

#### 2.4.1 DNA isolation

The Biobank of the Virgen de las Nieves University Hospital, which is part of the Biobank of the Andalusian Public Health System, provided DNA samples isolated from saliva or blood. Saliva samples were collected in 50 mL BD Falcon conical tubes (BD, Plymouth, United Kingdom). Blood samples were collected in 3 mL BD Vacutainer^®^ tubes with EDTA K3 as an anticoagulant. DNA extraction was performed using the QlAamp DNA Mini extraction kit (Qiagen GmbH, Hilden, Germany), following the specifications provided by the manufacturer for DNA purification from saliva or blood. Purified DNA samples were stored at −80°C in the Biobank of the Virgen de las Nieves University Hospital. DNA concentration and purity were measured with the NanoDrop 2000™ UV-visible spectrophotometer using the ratio of the absorbance at 260/280 and 260/230.

#### 2.4.2 Genotyping and quality control

The 13 polymorphisms, shown in **Table 1**, were determined by real-time polymerase chain reaction for allele discrimination using TaqMan^®^ probes (ABI Applied Biosystems, Quant Studio 3 Real-Time PCR System, 96 wells), following the manufacturer’s instructions. The polymorphisms *VDR*-Bsml (rs1544410), *CYP27B1* rs703842, and *CYP27B1* rs3782130 were analyzed using an assay customized by ThermoFisher Scientific (Waltham, Massachusetts, United States) encoded as AN324M4, AN9HX2K, and ANPRYR9, respectively. Sanger sequencing was performed in 10% of the samples and used to confirm of the results. Real-time PCR and Sanger sequencing were performed in the Pharmacogenetics Unit of the Virgen de las Nieves University Hospital and the Department of Biochemistry and Molecular Biology II of the University of Granada. The criteria for SNPs quality control were: 1) missing genotype rate per SNP < 0.05; 2) minor allele frequency > 0.01; 3) p-value > 0.05 in Hardy Weinberg equilibrium test; 4) missing genotype rate between cases and controls < 0.05.

**Table 1:** Studied single nucleotide polymorphisms and TaqMan^®^ ID.

Gene (Chromosome location)	Location, SNP	dbSNP ID	Assay ID
*VDR* (12q13.11)	Intron 8, G > A	rs1544410 (BsmI)	AN324M4
	Intron 1, G > A	rs11568820 (Cdx-2)	C:_2880808_10
	Exon 2, C > T	rs2228570 (FokI)	C:12060045_20
	Intron 8, C > A	rs7975232 (ApaI)	C:28977635_10
	Exon 9, T > C	rs731236 (TaqI)	C:_2404008_10
*CYP27B1* (12q14.1)	Intron 6, A > G	rs4646536	C:25623453_10
	Promotor 5’, G > C	rs3782130	ANGZRHH
	5’UTR, T > G 3’UTR, A > G	rs10877012 rs703842	C:26237740_10 ANH6J3F
*CYP24A1* (20q13.2)	Exon 6, G > A	rs6068816	C:25620091_20
	3’UTR, G > C	rs4809957	C:_3120981_20
*GC* (4q13.3)	Exon 11, T > G	rs7041	C:_3133594_30
*CYP2R1* (11p15.2)	5’UTR, A > G	rs10741657	C:_2958430_10

UTR, untranslated region.

### 2.5 Statistical analysis

Cases and controls were matched by age and sex with the propensity score matching method (1:3) using the RStudio Software (18). The descriptive analysis of the sociodemographic and clinical variables was performed using the statistical program R 4.2.0. The quantitative variables were expressed as mean (± standard deviation) for variables meeting normality criteria and as median (p50) and percentiles (p25 and p75) for the variables that did not follow a normal distribution. Normality was confirmed using the Kolmogorov-Smirnov test for a sample size larger than 50.

The analysis of genetic variants was performed using PLINK 1.9, the open-source whole genome association analysis toolset (19). The Hardy-Weinberg equilibrium, haplotype frequency, and linkage disequilibrium (LD) were determined, the Lewontin D prime coefficients (D’) and the disequilibrium coefficient (R2) were used to determine LD. These variables calculation is based on fundamental principles of population genetics. The analysis consists in the Chi-square test considering the allelic frequency in the study population. The LD was performed with the Haploview 4.2 software and the analysis of haplotypes with SNPStats, a web tool for analyzing association (20, 21).

Bivariate analysis of the association between COPD risk and vitamin D polymorphisms was performed with multiple models using Pearson’s Chi-square test and Fisher’s exact test (at expected frequencies below 5%) to calculate the adjusted probability ratio (OR) and the 95% confidence interval (95%CI).

A multivariate logistic regression analysis was performed with all the variables found significant in the bivariate logistic regression model. The Benjamini-Hochberg method was used to control the false discovery rate (FDR) when performing multiple comparisons. Unconditional multiple logistic regression models (genotypic, dominant, and recessive) were considered to determine the influence of possible confounding variables on COPD risk. All tests were bilateral, with a significance level of p < 0.05. Tests were performed with PLINK and the statistical program R 4.2.0 (R Foundation for Statical Computing, Vienna, Austria) (22, 23).

## 3 Results

### 3.1 Patient characteristics

A total of 608 individuals of Caucasian origin were included in the study: 152 COPD cases and 456 controls. **Supplementary Table S1** describes their clinical, sociodemographic, and pathological characteristics.

In the case group, the median age was 65 (59–71) years, 78.9% (120/152) were men, 63.8% (97/152) were former smokers, while 17.8% (27/152) were smokers. Most patients were overweight or obese, 44.7% (68/152) and 40.8% (62/152), respectively. Prior to diagnosis, 38.8% (59/152) had emphysema, 51.9% (79/152) chronic bronchitis, 28.9% (44/152) bronchial hyperreactivity, 84.2% (128/152) dyspnea, 73.0% (111/152) exacerbation, 69.7% (106/152) respiratory infection and 10.5% (16/152) HTP. Median saturation of O_2_ was 91 (78-94) mg/day, and pulmonary function expressed as the median of FEV1 (%) was 64 (47-73).

The control group had a mean age of 64 (54-72) years, and 78.9% (360/456) were men. 21.7% (99/456) were smokers and 34.4% (157/456) were former smokers. Regarding BMI, most patients were overweight (36.6%, 167/456), and a high percentage were obese (35.5%, 162/307).

Statistically significant differences were found between cases and controls regarding smoking (p = 0.003, OR = 1.95, 95%CI =1.09-3.49, smoker vs. non-smoker; and p = 0.003, OR = 4.41, 95%CI = 2.79-7.16, ex-smoker vs. Non-smoker) and BMI (p = 0.003, OR = 2.35, 95%CI = 1.40-4.08, overweight vs. normal weight; and p = 0.003, OR = 2.21, 95%CI = 1.31-3.85, obesity vs. normal weight). No statistically significant differences were found in sex (p = 1), age (p = 0.058), and alcohol consumption (p = 0.059).

### 3.2 Genotype distribution

**Figure 1:**
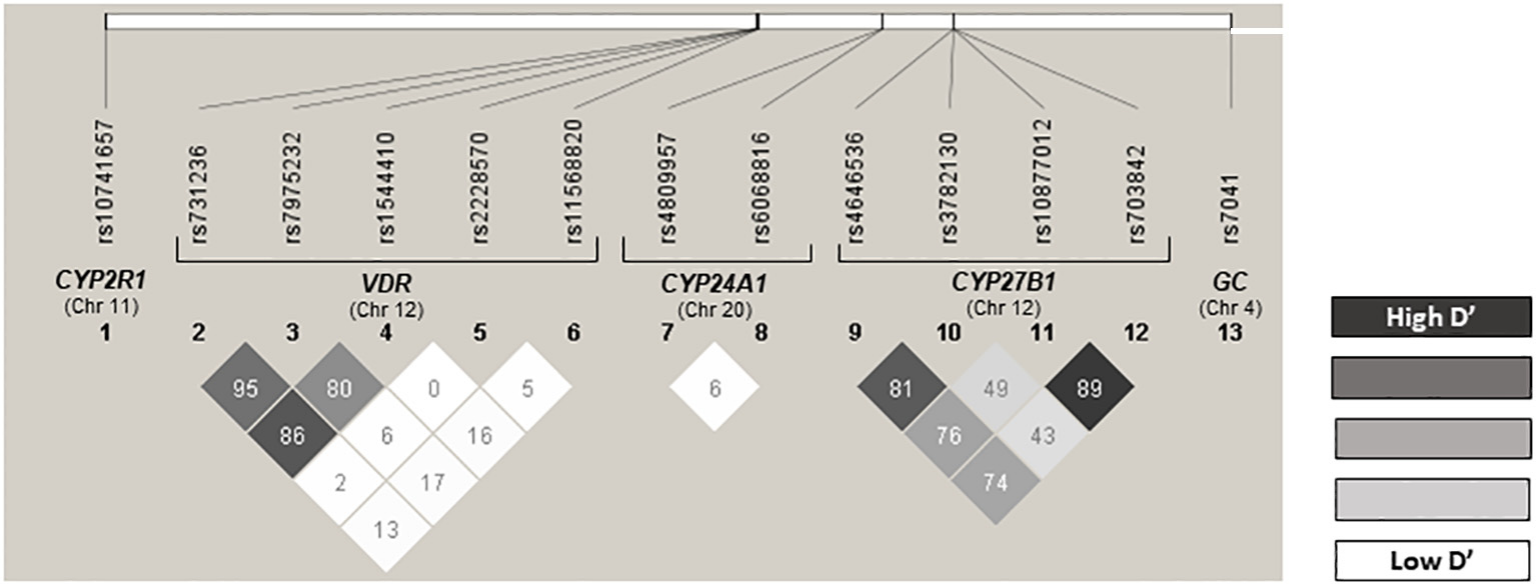
Linkage disequilibrium (LD). This figure shows the LD of the 13 SNPs included in this study, separated according to the gene. D' value higher than 0.7 means the SNPs are in LD.

All polymorphisms were successfully genotyped and showed a minor allele frequency (MAF) above 1%, so none were excluded from the analysis (**Supplementary Table S2**). The found genotypic frequencies were consistent with the expected values according to the Hardy-Weinberg equilibrium model (HDW), except for *CYP27B1* rs4646536 (p < 0.001), *CYP27B1* rs3782130 (p = 0.028), and *CYP24A1* rs6068816 (p < 0.001) in the control group and *VDR* FokI (rs2228570) (p = 0.021), *CYP27B1* rs3782130 (p < 0.001), and *CYP24A1* rs6068816 (p < 0.001) in the case group (**Supplementary Table S3**). No statistical differences were found between the MAF obtained and those described in the Iberian population for these variants: *CYP27B1* rs4646536 G allele: 0.386 vs. 0.290 (p = 0.151), *CYP27B1* rs3782130 T allele: 0.401 vs. 0.285 (p = 0.083) and *CYP24A1* rs6068816 A allele: 0.217 vs. 0.107 (p = 0.056) (24). The values of D’ and R2 are shown in **Supplementary Table S4**, and **Figure 1** shows the graph of LD. Polymorphism pairs *CYP27B1* rs10877012/rs703842 (D’ = 0.8940), *CYP27B1* rs4646536/3782130 (D’ = 0.8165), *VDR* rs731236/rs7975232 (D’ = 0.9507), *VDR* rs731236/rs1544410 (D’ = 0.8623), and *VDR* rs7975232/rs1544410 (D’ = 0.8030) showed strong LD (**Supplementary Table S4**, **Figure 1**). **Supplementary Table S5** shows the estimated frequencies of haplotypes. The most frequent haplotype was GCTAAGG (cumulative frequency = 0.2328; **Supplementary Table S5**), corresponding to SNPs rs11568820/rs7975232/rs731236/rs4646536/rs703842/rs3782130/rs10877012.

### 3.3 Influence of gene polymorphisms on COPD risk

The bivariate analysis considered the following models: Genotypic, allelic, recessive, dominant, and additive for all polymorphisms and the risk of developing COPD (**Supplementary Table S6**). The SNPs of the genes *CYP2R1* rs10741657, *CYP27B1* rs4646536, and *CYP27B1* rs703842 showed a significant association with the risk of developing COPD and the *VDR*-Fokl rs2228570 a tendency toward the statistical association (**Supplementary Table S6**).

For *CYP2R1* rs10741657 polymorphism, the dominant model revealed that patients with the A allele presented a higher risk of developing COPD (p = 0.018; OR = 1.59; 95% CI = 1.09-2.34, A vs. GG; **Table 2**). Allelic and additive models confirmed the association between the A allele and a higher COPD risk (p = 0.016, OR = 1.38, 95% CI = 1.05-1.82, A vs G; and p = 0.021, OR = 1.35, 95% CI = 1.05-1.75; **Table 2**). Moreover, in the genotypic model, a tendency toward statistical significance was found for A allele carriers (p = 0.053, OR = 1.74, 95% CI = 1.01-2.98, AA vs. GG, and p = 0.053, OR = 1.53, 95% CI = 1.02-2.32, AG vs. GG; **Table 2**).

**Table 2:** Influence of *CYP2R1* rs10741657, *CYP27B1* rs4646536, and *CYP27B1* rs703842 gene polymorphisms on the risk of COPD.

Models	Genotype	Cases (n (%))	Controls (n (%))	*p*-value^a^	OR (CI95%)
*CYP2R1* rs10741657					
Genotypic	AA	28 (18.4)	64 (14.0)	0.053	1.74 (1.01-2.98)
	AG	73 (48.0)	189 (41.5)		1.53 (1.02-2.32)
	GG	54 (33.6)	203 (44.5)		1
Dominant	AA + AG	101 (66.4)	253 (55.5)	0.018	1.59 (1.09-2.34)
	GG	51 (33.6)	203 (44.5)		1
Recessive	AA	28 (18.4)	64 (14.0)	0.191	
	AG + GG	124 (81.6)	392 (86.0)		
Allelic	A	129 (42.4)	317 (34.8)	0.016	1.38 (1.05-1.82)
	G	175 (57.6)	595 (65.2)		1
Additive	–	–	–	0.021	1.35 (1.05-1.75)
*CYP27B1* rs4646536					
Genotypic	GG	13 (8.5)	84 (18.4)	<0.001	1
	AG	50 (32.9)	176 (38.6)		1.84 (0.97-3.69)
	AA	89 (58.6)	196 (43.0)		2.93 (1.60-5.76)
Dominant	GG + AG	63 (41.4)	260 (57.0)	<0.001	1
	AA	89 (58.6)	196 (43.0)		1.87 (1.29-2.73)
Recessive	GG	13 (8.5)	84 (18.4)	0.004	1
	AG + AA	139 (91.5)	372 (81.6)		2.41 (1.35-4.66)
Allelic	G	76 (25.0)	344 (37.7)	0.054	1
	A	228 (75.0)	568 (62.3)		1.82 (1.35-2.47)
Additive	–	–	–	<0.001	1.67 (1.28-2.21)
*CYP27B1* rs703842					
Genotypic	GG	7 (4.6)	32 (7.0)	0.041	1
	AG	46 (30.3)	180 (39.5)		1.16 (0.51-3.03)
	AA	99 (65.1)	244 (53.5)		1.85 (0.84-4.70)
Dominant	GG + AG	53 (34.9)	212 (46.5)	0.012	1
	AA	99 (65.1)	244 (53.5)		1.62 (1.11-2.39)
Recessive	GG	7 (4.6)	32 (7.0)	0.293	
	AG + AA	145 (95.4)	424 (93.0)		
Allelic	G	60 (19.7)	244 (26.8)	0.014	1
	A	244 (80.3)	668 (73.2)		1.48 (1.07-2.08)
Additive	–	–	–	0.015	1.48 (1.09-2.06)

a p-value for χ² test. Shade means the value is significant. OR, Odds ratio; CI, Confidence interval.

In the *CYP27B1* rs4646536 SNP, the genotypic and dominant models showed that the presence of the AA genotype was associated with a higher risk of developing COPD (p < 0.001, OR = 2.93, 95% CI = 1.60-5.76, AA vs. GG, and p < 0.001, OR = 1.87, 95% CI = 1.29-2.73, AA vs. G; **Table 2**). In the recessive model, it was found that A allele carriers had a higher risk of COPD (p = 0.004, OR = 2.41, 95% CI = 1.35-4.66, A vs. GG; **Table 2**). The additive model confirmed this association (p < 0.001, OR = 1.67, 95% CI = 1.28-2.21; **Table 2**), and the allelic model revealed a trend toward statistical significance in the same line (p = 0.054, OR = 1.82, 95% CI = 1.35-2.47, A vs. G; **Table 2**).

For the *CYP27B1* rs703842 polymorphism, the dominant model revealed that patients with the AA genotype had a higher risk of developing COPD (p = 0.012, OR = 1.62, 95% CI = 1.11-2.39, AA vs. G; **Table 2**). Allelic and additive models confirmed the association of A allele with a higher risk of disease (p = 0.014, OR = 1.48, 95% CI = 1.07-2.08, A vs. G, and p = 0.015, OR = 1.48, 95% CI = 1.09-2.06, respectively, **Table 2**).

A multivariate analysis was performed for each genetic model (**Table 3**). The genotypic model confirmed a higher risk of COPD in patients with obesity (p = 0.017), overweight (p = 0.015), smokers (p = 0.016), former smokers (p < 0.001), and carriers of the *CYP2R1* rs10741657-AA genotypes (p = 0.028) and *CYP27B1* rs4646536-AA (p = 0.004). The dominant model revealed a higher risk of COPD in patients with obesity (p = 0.014), overweight (p = 0.012), smokers (p = 0.024), former smokers (p < 0.001) and in carriers of the *CYP27B1* rs4646536-AA genotype (p = 0.008). In consistency with these results, in the recessive model, obesity (p = 0.016), overweight (p = 0.013), smokers (p = 0.014), former smokers (p < 0.001), and *CYP27B1* rs4646536-A allele carriers (p = 0.013) were associated with a higher COPD risk. Finally, the additive model estimated that patients who were smokers (p = 0.017), former smokers (p < 0.001), overweight (p = 0.014), or obese (p = 0.016), and carriers of *CYP2R1* rs10741657-A (p = 0.027) and *CYP27B1* rs4646536-A (p = 0.020) alleles were associated with an increased risk of COPD. After applying the FDR adjustment, it is remarkable that all the variables in our study maintained their significance, thus reinforcing the robustness and reliability of our results (**Table 3**).

**Table 3:** Influence of clinical characteristics and *CYP2R1* (rs10741657) and *CYP27B1* (rs4646536) gene polymorphism on risk of COPD.

	Genotypic				Dominant		Recessive		Additive	
	DD vs. dd		Dd vs. dd		DD + Dd vs. dd		DD vs. Dd + dd		DD = 0, Dd = 1, dd = 2	
	*p*-value	OR (95%IC)	*p*-value	OR (95%IC)	*p*-value	OR (95%IC)	*p*-value	OR (95%IC)	*p*-value	OR (95%IC)
Current smoker	0.016 0.020^a^	2.08 (1.14-3.77)	0.016 0.026^a^	2.08 (1.14-3.77)	0.024 0.029 ^a^	1.98 (1.09-3.57)	0.014 0.019 ^a^	2.08 (1.15-3.76)	0.017 0.024 ^a^	2.06 (1.13-3.74)
Former smokers	<0.001 <0.001 ^a^	4.21 (2.63-6.91)	<0.001 <0.001 ^a^	4.21 (2.63-6.91)	<0.001 <0.001 ^a^	4.1 (2.58-6.69)	<0.001 <0.001 ^a^	4.25 (2.68-6.94)	<0.001 <0.001 ^a^	4.17 (2.62-6.83)
Obesity	0.017 0.020 ^a^	1.99 (1.14-3.56)	0.017 0.026 ^a^	1.99 (1.14-3.56)	0.014 0.021 ^a^	2.01 (1.16-3.58)	0.016 0.019 ^a^	1.99 (1.15-3.76)	0.016 0.024 ^a^	1.99 (1.15-3.56)
Overweight	0.015 0.020 ^a^	2 (1.16-3.55)	0.015 0.026 ^a^	2 (1.16-3.55)	0.012 0.021 ^a^	2.04 (1.19-3.6)	0.013 0.019 ^a^	2.01 (1.17-3.55)	0.014 0.024 ^a^	2 (1.62-3.55)
*CYP2R1* rs10741657	0.028 0.028 ^a^	1.9 (1.06-3.36)	0.200 0.200 ^a^	1.32 (0.86-2.05)	0.066 0.066 ^a^	1.45 (0.98-2.19)	0.067 0.067 ^a^	1.62 (0.95-2.72)	0.027 0.027 ^a^	1.37 (1.04-1.81)
*CYP27B1* rs4646536	0.004 0.012 ^a^	2.6 (1.38-5.22)	0.096 0.115 ^a^	1.79 (0.92-3.69)	0.008 0.021 ^a^	1.69 (1.15-2.5)	0.013 0.019 ^a^	2.24 (1.22-4.41)	0.020 0.024 ^a^	1.56 (1.18-2.08)

a *p*-value for Benjamini-Hochberg (FDR) correction. The table shows the results corresponding to the genetic variant associated with risk; therefore, for the SNP rs4646536, the reference allele for genotypic, dominant, and recessive models is the minor allele. Shade means the value is significant for t test (p < 0.05).

The haplotype analysis showed an association (p < 0.0001) between the haplotype CCTAGGT and an increased risk of COPD (**Table 4**). In contrast, the CACAACG haplotype was associated (p = 0.035) with a lower risk of developing the disease (**Table 4**). These haplotypes correspond to the SNPs *VDR* (rs1544410, rs7975232, rs731236), and *CYP27B1* (rs4646536, rs703842, rs3782130, rs10877012).

**Table 4:** Association of *VDR* and *CYP27B1* haplotypes with risk of COPD.

	*VDR* rs11568820	*VDR* rs7975232	*VDR* rs731236	*CYP27B1* rs4646536	*CYP27B1* rs703842	*CYP27B1* rs3782130	*CYP27B1* rs10877012	Freq	OR (95% CI)	p-value
1	C	C	T	A	A	G	G	0.2309	1.00	‐‐‐
2	C	A	C	A	A	G	G	0.1356	0.73 (0.42 - 1.26)	0.260
3	T	A	C	A	A	G	G	0.075	1.16 (0.60 - 2.25)	0.660
4	C	A	C	G	G	C	T	0.0682	1.27 (0.63 - 2.57)	0.510
5	C	A	T	A	A	G	G	0.0545	0.80 (0.41 - 1.54)	0.500
6	T	C	T	A	A	G	G	0.0516	1.69 (0.60 - 4.81)	0.320
7	C	C	T	G	A	C	G	0.0453	1.98 (0.85 - 4.60)	0.110
8	C	C	T	G	G	C	T	0.0412	0.75 (0.27 - 2.05)	0.570
9	C	A	C	G	A	C	G	0.0363	1.91 (0.75 - 4.83)	0.170
10	C	A	T	G	G	C	T	0.017	1.17 (0.27 - 4.99)	0.840
11	C	A	C	G	G	G	T	0.0161	2.54 (0.53 - 12.25)	0.250
12	T	A	T	A	A	G	G	0.0153	0.74 (0.21 - 2.66)	0.650
13	T	A	C	G	A	C	G	0.0152	1.75 (0.40 - 7.67)	0.460
14	T	C	T	G	G	C	T	0.0145	1.13 (0.18 - 7.15)	0.890
15	T	A	C	G	G	C	T	0.0137	0.57 (0.07 - 4.61)	0.600
16	C	A	C	A	A	C	G	0.0121	0.18 (0.04 - 0.88)	0.035
17	C	C	T	A	G	G	T	0.0105	1.1e^46^ (1.1e^46 1^.1e^46^)	<0.0001
Rare	*	*	*	*	*	*	*	0.1470	1.17 (0.71-1.94)	0.540

Global haplotype association p-value: 0.008

Freq, haplotype frequency.

The symbol * means the haplotype are rare and there is no symbol for them.

## 4 Discussion

COPD is one of the leading public health problems worldwide, being the third leading cause of death and the seventh leading cause of poor health worldwide. To date, the main risk factor is exposure to certain environmental and occupational pollutants, along with tobacco use (25). In addition, low serum levels of vitamin D have been reported to influence the risk of developing COPD (26). Moreover, a recent meta-analysis has reported that vitamin D supplementation in patients with asthma and COPD improves the disease state (5). However, few studies evaluate the impact of the genetic variants involved in vitamin D metabolism on the onset and development of the disease, focusing on the *GC* gene (27, 28). The relevance of this study lies in the higher coverage of the vitamin D metabolic pathway in order to study association of SNPs related this metabolism with COPD risk. Thus, we included *GC*, *CYP2R1*, *CYP27B1*, *CYP24A1*, and *VDR* gene polymorphisms.

The *CYP2R1* gene encodes for the 25-hydroxylase enzyme, which catalyzes the hydroxylation of the two inactive isoforms of vitamin D in position 25, mainly in the liver. As a result of this reaction, calcidiol, the main metabolite of vitamin D in the blood, is obtained (29, 30). After evaluating the influence of *CYP2R1* rs10741657 polymorphism on COPD risk, we found that those carriers of the A allele may have a higher risk of developing this condition than carriers of the GG genotype (**Table 4**). To date, no studies have been reported evaluating the potential of this SNP in the COPD risk, but these results suggest that this SNP may play an important role in genetic susceptibility to COPD. Moreover, its effect on the risk of developing other lung diseases, such as asthma, has been determined, and inconsistent results have been found. First, the study conducted by Lahmar et al. (2018) in a Caucasian population (Tunisia, 154 cases/154 controls) reported a strong trend among A allele carriers and the development of asthma (p = 0.052; OR = 1.41; 95% CI = 0.99-2.00; A vs GG), in consistency with our results (31). In contrast, a study conducted in another Caucasian population (Spain, 221 cases/442 controls) found no significant association between polymorphism rs10741657 and asthma risk (p = 0.603) (10). In addition, it has been observed that the SNP *CYP2R1* rs10741657 influences blood levels of vitamin D, where in a meta-analysis conducted in 2018 by Duan et al. with a total of 52,417 participants, the authors found that, in the Caucasian population, the presence of AG and AA genotypes leads to an alteration of 25(OH)D levels under the genotypic model (I^2^ = 69.2%, SEM = −1.27, 95% CI = −2.32 - −0.23) (32).

The *CYP27B1* gene encodes for 1-α-hydroxylase, an enzyme responsible for the hydroxylation of calcidiol at position 1, resulting in calcitriol (the active form of vitamin D) (28, 29). This gene is mainly expressed in the kidney, although 1-α-hydroxylase has also been found in immune response cells related to the risk and severity of COPD (dendritic cells, macrophages, and B and T lymphocytes) (33). We evaluated the effect of 4 polymorphisms located in this gene (rs4646536, rs703842, rs10877012, and rs3782130) on the risk of COPD, considering that *CYP27B1* variants could affect the availability of the active form of vitamin D in the body. Our results revealed that patients with the *CYP27B1* rs4646536-A allele may have a higher risk of COPD (**Table 4**). The correlation of this SNP with the risk of developing COPD has not been studied previously. However, the result of our study would support the hypothesis that *CYP27B1* genetic alterations may impact vitamin D homeostasis and, therefore, influence the predisposition to COPD. Our results are consistent with the reported by a study conducted in an Asian population (China), including 143 cases of childhood bronchial asthma and 143 controls, where they showed that the G allele of *CYP27B1* rs4646536 was associated with a lower risk of developing asthma (p < 0.05, OR = 0.69, 95% CI = 0.49-0.98) (34). Furthermore, we found that carriers of A allele for *CYP27B1* rs703842 showed a higher risk of COPD. However, this effect was not found in the multivariate analysis. No reports were found in the scientific literature about the effect of this SNPs in relation to COPD risk. However, the results obtained in the present study are consistent with those found in a study with 221 cases/442 controls in a Caucasian population (Spain), where no association was found between *CYP27B1* rs703842 and susceptibility to asthma development (p = 0.522) (10). We found no association of *CYP27B1* rs10877012 and rs3782130 polymorphisms with COPD risk in our study population. The effect on COPD risk of these SNPs has not been previously reported in the literature.

VDR is a nuclear receptor to which calcitriol binds, allowing it to form a complex with the RXR and translocate into the nucleus to act as a transcription factor. This receptor is encoded by the *VDR* gene, which is expressed broadly in different cells in the body, including immune cells (28, 29, 35). In our study, we have evaluated the effect of the five most relevant polymorphisms reported for this gene (BsmI, FokI, TaqI, ApaI, and Cdx2) on susceptibility to COPD. A trend between *VDR* FokI C allele and COPD risk has been observed in the recessive model of bivariate analysis (p = 0.063, **Supplementary Table S6**). Although no previous studies have been reported linking this SNP to the risk of COPD, our results are consistent with what has been reported regarding other lung diseases. In a meta-analysis including 18 mixed population studies (Egypt, Turkey, Chile, China, Ireland, Greece, Tunisia, Cyprus, and the USA), the *VDR* FokI T allele was associated with a decrease in asthma risk in the dominant model (p = 0.016, OR = 0.77, 95%CI = 0.63-0.95, TT+CT vs. CC) (36). In our study, no association was found between the rest of the *VDR* SNP (BsmI, TaqI, ApaI, and Cdx2) and the risk of developing COPD.

One of the most studied polymorphisms in the *GC* gene is rs7041, located at exon 11 (10). This SNP has been shown to affect 25(OH)D levels (37). No association between the *GC* rs7041 polymorphism and COPD risk was found in our study. However, the literature shows contradictory results, possibly linked to ethnic differences, sample size, and applied methodology (10, 38).

The enzyme 24-hydroxylase, encoded by the *CYP24A1* gene, is responsible for the inactivation of vitamin D, with initial hydroxylation of 1α,25(OH)2D3 and 25(OH)D3 mainly in C24 to produce 1α,24,25(OH)3D3 and 24 R,25(OH)2D3, respectively (39, 40). Two of the most researched SNPs in this gene are rs4809957 and rs6068816. After evaluating the potential of these SNPs on COPD risk under multiple genetic models, we found no statistically significant association. To date, no previous studies have been reported that relate these polymorphisms to COPD.

This project has some typical limitations of retrospective studies. The main one is the limited sample size, which may prevent us from finding certain associations. Furthermore, the *CYP2R1* rs10741657 and *CYP27B1* rs4646536 SNPs maintained their association with COPD after using the Benjamini-Hochberg correction to avoid false positive associations. Moreover, serum vitamin D levels could not be included in the study due to the absence of this information in the medical records of the subjects. Regarding the strengths of the study, the homogeneity of the included population is noteworthy because all cases have been diagnosed in the Virgen de las Nieves University Hospital and all controls belong to the same geographical area. In addition, the cases and controls have been matched by age and sex, thus increasing the uniformity of both samples.

The results of the present study indicate that individuals with *CYP2R1* rs10741657 A allele and *CYP27B1* rs4646536 A allele may have a higher risk of developing COPD. This is the first study in which SNPs located in genes related to the complete metabolic pathway of vitamin D were evaluated. Therefore, the results obtained should be taken with caution because of the limitations of the study and the need for larger evidence for its clinical use. Furthermore, these SNPs have been associated with other chronical diseases such as asthma, cardiovascular diseases, arthritis, obesity and type II diabetes, which highlights the possible role of vitamin D in a wide range of molecular mechanisms (11, 41–44).

This is a pioneering study of the main SNPs of the vitamin D pathway and their association with the COPD risk. Significant results advancing the understanding of this highly prevalent lung disease are found. Through a thorough analysis of genetic variability in two key genes, *CYP2R1* and *CYP27B1*, we have identified a possible relevant relationship, confirming that the genetic variant rs10741657 of the *CYP2R1* gene, represented by the A allele, and the genetic variant rs4646536 of the *CYP27B1* gene, also represented by A allele, along with smoking habit, are significantly associated with an increased risk of COPD.

These results provide a new perspective on the underlying genetics of COPD and underline the importance of the interaction between genetics and environmental factors in the pathogenesis of this disease. In addition, these findings can influence the identification of subgroups of patients at risk and, therefore, the prevention and personalized management of COPD. However, further research is needed to validate these results and explore potential therapeutic implications in COPD.

## Data Availability

The datasets presented in this study can be found in online repositories. The names of the repository/repositories and accession number(s) can be found here: https://doi.org/doi:10.5061/dryad.fbg79cp56.
