# Supplementary material for: Vitamin D metabolism-related single nucleotide polymorphisms in Chronic Obstructive Pulmonary Disease risk
Source: Front Endocrinol (Lausanne). 2024 Nov 8;15:1445712. doi: 10.3389/fendo.2024.1445712 (PMC11581940; doi:10.3389/fendo.2024.1445712)
Supplement: Supplementary file 1 [file DataSheet1.pdf]

frontiers

Supplementary Material

1 Supplementary Figures and Tables

S1 Table. Clinico-pathologic characteristics of asthma cases and controls.

	Cases		Controls		χ^2	p-value	OR	95%CI
	N	n (%)	N	n (%)				
Sex	152		456					
Female		32 (21.1)		96 (21.1)	0	1		
Male		120 (78.9)		360 (78.9)				
Age	152	65 (59-71)	456	64 (54-72)		0.058		
Smoking habits	152		456					
Current smoker		27 (17.8)		99 (21.7)	44.094	0.003	1.95	1.09-3.49
Former smoker		97 (63.8)		157 (34.4)			4.41	2.79-7.16
Non-smoker		28 (18.4)		200 (43.9)			1	
Body Mass Index (kg/m^2)	152		456					
Normal weight		22 (14.5)		127 (27.9)	11.123	0.003	1	
Overweight		68 (44.7)		167 (36.6)			2.35	1.40-4.08
Obesity		62 (40.8)		162 (35.5)			2.21	1.31-3.85
Alcohol consumption	152		456					
Drinker		40 (26.3)		126 (27.6)	5.651	0.059		
Former drinker		14 (9.2)		19 (4.2)				
Non-drinker		98 (64.5)		311 (68.2)				
COPD phenotype	152							
Sharpening		59 (38.8)						
Non-acute		56 (36.8)						
Asthma-COPD-overlap		37 (24.4)						
COPD severity	152							
GOLD1 (mild)		15 (9.9)						
GOLD2 (moderate)		92 (60.5)						
GOLD3 (severe)		40 (26.3)						
GOLD4 (very severe)		5 (3.3)						
Emphysema	152							
Yes		59 (38.8)						
No		93 (61.2)						
Chronic bronchitis	152							
Yes		79 (52.0)						
No		73 (48.0)						

Bronchial hyperresponsiveness	152	
Yes		44 (28.9)
No		108 (71.1)
O_2 saturation	152	91 (77-94)
Respiratory insufficiency	152	
Yes		69 (45.4)
No		83 (54.6)
Dyspnea	152	
Yes		128 (84.2)
No		24 (15.8)
Previous exacerbation	152	
Yes		111 (73.0)
No		41 (27.0)
%FEV1	152	64 (47-73)
Respiratory infection	152	
Yes		106 (69.7)
No		46 (30.3)
Pulmonary hypertension	152	
Yes		16 (10.5)
No		136 (89.5)
Osteoporosis	152	
Yes		23 (15.1)
No		129 (84.9)
Obstructive sleep apnea	152	
Yes		27 (17.8)
No		125 (82.2)

S2 Table. Minor allele frequencies of SNPs.

Chr	SNP	Minor Allele	Major Alelle	MAF
4	rs7041	A	C	0.4416
11	rs10741657	A	G	0.3668
12	rs731236	C	T	0.4112
12	rs7975232	C	A	0.4671
12	rs1544410	A	G	0.4416
12	rs2228570	T	C	0.3281
12	rs11568820	A	G	0.2426
12	rs4646536	G	A	0.3454
12	rs3782130	C	G	0.3372

12	rs10877012	T	G	0.2442
12	rs703842	G	A	0.25
20	rs4809957	G	A	0.2294
20	rs6068816	T	C	0.1472

Chr: Chromosome; MAF: Minor allele frequency

S3 Table. Hardy-Weinberg Equilibrium.

Chr	SNP	Sample	Minor allele	Major allele	Genotypic count	Heterozygosity observed	Expected heterozygosity	p-value
4	rs7041	ALL	A	C	111/315/182	0.5181	0.4932	0.2494
4	rs7041	AFF	A	C	30/75/47	0.4934	0.4937	1
4	rs7041	UNAFF	A	C	81/240/135	0.5263	0.493	0.1832
11	rs10741657	ALL	A	G	92/262/254	0.4309	0.4645	0.0806
11	rs10741657	AFF	A	G	28/73/51	0.4803	0.4886	0.8684
11	rs10741657	UNAFF	A	G	64/189/203	0.4145	0.4535	0.0639
12	rs731236	ALL	C	T	105/290/213	0.477	0.4842	0.7376
12	rs731236	AFF	C	T	31/68/53	0.4474	0.4895	0.32
12	rs731236	UNAFF	C	T	74/222/160	0.4868	0.4822	0.9226
12	rs7975232	ALL	C	A	136/296/176	0.4868	0.4978	0.6249
12	rs7975232	AFF	C	A	36/68/48	0.4474	0.4969	0.2526
12	rs7975232	UNAFF	C	A	100/228/128	0.5	0.4981	1
12	rs1544410	ALL	T	C	128/281/199	0.4622	0.4932	0.119
12	rs1544410	AFF	T	C	33/68/51	0.4474	0.493	0.2527
12	rs1544410	UNAFF	T	C	95/213/148	0.4671	0.4932	0.2557
12	rs2228570	ALL	T	C	74/251/283	0.4128	0.4409	0.1182
12	rs2228570	AFF	T	C	25/56/71	0.3684	0.4542	0.0207
12	rs2228570	UNAFF	T	C	49/195/212	0.4276	0.4361	0.6685
12	rs11568820	ALL	A	G	34/227/347	0.3734	0.3675	0.7415
12	rs11568820	AFF	A	G	9/48/95	0.3158	0.3399	0.3485
12	rs11568820	UNAFF	A	G	25/179/252	0.3925	0.3761	0.3855
12	rs4646536	ALL	G	A	97/226/285	0.3717	0.4522	1,52E-02
12	rs4646536	AFF	G	A	13/50/89	0.3289	0.375	0.1321
12	rs4646536	UNAFF	G	A	84/176/196	0.386	0.4698	0.0001
12	rs3782130	ALL	C	G	89/232/287	0.3816	0.447	0.0003
12	rs3782130	AFF	C	G	26/49/77	0.3224	0.4437	0.0009
12	rs3782130	UNAFF	C	G	63/183/210	0.4013	0.448	0.02806
12	rs10877012	ALL	T	G	34/229/345	0.3766	0.3692	0.6616
12	rs10877012	AFF	T	G	6/51/95	0.3355	0.3286	1
12	rs10877012	UNAFF	T	G	28/178/250	0.3904	0.3815	0.7127
12	rs703842	ALL	G	A	39/226/343	0.3717	0.375	0.829
12	rs703842	AFF	G	A	7/46/99	0.3026	0.3168	0.6078
12	rs703842	UNAFF	G	A	32/180/244	0.3947	0.3919	1
20	rs4809957	ALL	G	A	33/213/362	0.3503	0.3536	0.8188
20	rs4809957	AFF	G	A	12/47/93	0.3092	0.358	0.1104
20	rs4809957	UNAFF	G	A	21/166/269	0.364	0.3521	0.5091
20	rs6068816	ALL	T	C	26/127/455	0.2089	0.2511	0.0001
20	rs6068816	AFF	T	C	5/28/119	0.1842	0.2188	0.05964
20	rs6068816	UNAFF	T	C	21/99/336	0.2171	0.2614	0.0009

Chr: Chromosome; AFF: affected (cases); UNAFF: unaffected (controls).

S4 Table. Linkage disequilibrium

Chr	BP	SNP	Chr	BP	SNP	R2	D’
12	47844974	rs731236	12	47845054	rs7975232	0.533627	0.950721
12	47844974	rs731236	12	47846052	rs1544410	0.581797	0.862326
12	47845054	rs7975232	12	47846052	rs1544410	0.424857	0.803005
12	57764205	rs4646536	12	57768115	rs3782130	0.541685	0.81654
12	57768302	rs10877012	12	57768956	rs703842	0.763368	0.894043
Chr: Chromosome; BP: Physical position (base-pair)							

S5 Table. Frequencies of the *CYP27B1* and *VDR* haplotypes.

	VDR rs11568820	*VDR* rs7975232	*VDR* rs731236	*CYP27B1* rs4646536	*CYP27B1* rs703842	*CYP27B1* rs3782130	*CYP27B1* rs10877012	Total	Case group	Control group	Cumulative frequency
1	G	C	T	A	A	G	G	0.2328	0.2524	0.2216	0.2328
2	G	A	C	A	A	G	G	0.1367	0.1817	0.1245	0.3694
3	A	A	C	A	A	G	G	0.0732	0.0745	0.0714	0.4426
4	G	A	C	G	G	C	T	0.066	0.061	0.0667	0.5086
5	G	A	T	A	A	G	G	0.0546	0.0713	0.0503	0.5632
6	A	C	T	A	A	G	G	0.0496	0.0363	0.0593	0.6128
7	G	C	T	G	A	C	G	0.0451	0.0164	0.0559	0.6579
8	G	C	T	G	G	C	T	0.0423	0.0539	0.0371	0.7002
9	G	A	C	G	A	C	G	0.0363	0.0132	0.0447	0.7365
10	A	A	T	A	A	G	G	0.0164	0.0218	0.0114	0.7529
11	G	A	T	G	G	C	T	0.0162	0.0176	0.016	0.7691
12	G	A	C	G	G	G	T	0.0162	0.0059	0.019	0.7853
13	A	A	C	G	G	C	T	0.016	0.0213	0.0129	0.8013
14	A	C	T	G	G	C	T	0.015	0.017	0.0121	0.8163
15	A	A	C	G	A	C	G	0.015	0.0066	0.0183	0.8312
16	G	A	C	A	A	C	G	0.0137	0.0327	0.0045	0.8449
17	G	C	T	A	G	G	T	0.01	NA	0.0145	0.8549
18	G	C	T	A	A	C	G	0.0098	0.0236	0.009	0.8648
19	A	C	T	G	A	C	G	0.0093	0.0033	0.0113	0.8741
20	G	A	C	A	G	G	T	0.0093	NA	0.0138	0.8834
21	A	A	T	G	G	C	T	0.0081	NA	0.0131	0.8915
22	G	A	T	G	A	C	G	0.0079	NA	0.0106	0.8993
23	G	C	T	A	G	G	G	0.0075	0.0033	0.0098	0.9069
24	A	C	T	A	A	C	G	0.0073	0.0196	0.0012	0.9142
25	G	C	T	G	G	G	T	0.0062	0	0.0077	0.9203
26	G	A	T	A	A	C	G	0.0055	0.0166	0	0.9258
27	A	A	T	G	A	C	G	0.0054	NA	0.0074	0.9312
28	G	C	T	G	A	G	G	0.004	NA	0.0058	0.9352
29	G	A	T	A	G	G	T	0.0039	NA	0.0055	0.9391
30	G	C	T	G	G	G	G	0.0036	0.0034	0.0029	0.9426
31	A	C	T	G	G	G	T	0.0034	NA	0.0044	0.946
32	G	A	C	A	G	G	G	0.0033	0.0034	0.0029	0.9493

33	A	A	C	A	G	G	T	0.0031	NA	0.0041	0.9524
34	G	C	C	A	A	G	G	0.0029	0.0058	0.0013	0.9554
35	A	C	T	A	A	G	T	0.0028	NA	0.0028	0.9582
36	G	C	T	G	G	C	G	0.0027	NA	0.0035	0.9609
37	A	A	C	G	G	C	G	0.0026	NA	0.0034	0.9635
38	G	A	T	G	G	G	G	0.0026	NA	0.0036	0.9661
39	A	A	T	G	G	G	T	0.0024	NA	0.003	0.9684
40	G	A	T	G	G	G	T	0.0023	NA	0.003	0.9707
41	G	C	T	G	A	C	T	0.0023	0.0087	NA	0.973
42	G	C	T	G	A	G	T	0.0017	NA	0.0022	0.9748
43	G	A	T	A	A	G	T	0.0017	0.0033	NA	0.9765
44	A	A	T	A	A	G	T	0.0017	NA	0.0033	0.9782
45	A	A	C	A	A	C	G	0.0016	0.0037	0.0015	0.9797
46	G	A	T	G	A	C	T	0.0014	NA	0.0027	0.9812
47	G	A	C	G	A	C	T	0.0014	0.0036	NA	0.9826
48	A	C	T	A	G	G	T	0.0013	NA	0.002	0.9839
49	G	A	C	A	A	G	T	0.0012	NA	0.0015	0.9851
50	A	C	C	A	G	G	T	0.0012	NA	0.0013	0.9862
51	G	A	C	A	A	C	T	0.001	NA	0.0015	0.9873
52	G	A	C	G	A	G	G	9e-04	NA	0.0012	0.9882
53	G	C	C	G	A	C	G	9e-04	NA	0.0012	0.9892
54	G	A	C	G	G	G	G	9e-04	NA	0.0021	0.9901
55	G	A	C	G	A	G	T	9e-04	NA	0.0011	0.991
56	A	C	C	G	A	C	T	9e-04	0.0038	NA	0.9919
57	G	C	C	G	G	G	G	9e-04	0.0032	NA	0.9927
58	A	C	C	G	A	G	G	9e-04	NA	0.0011	0.9936
59	A	A	T	G	A	G	T	8e-04	NA	0.0011	0.9944
60	A	A	C	G	A	G	G	8e-04	NA	0.0012	0.9953
61	A	C	C	A	G	C	T	8e-04	NA	0.0011	0.9961
62	G	A	C	A	G	C	T	7e-04	NA	0.0013	0.9968
63	A	C	C	G	G	C	T	7e-04	0.0039	NA	0.9975
64	A	C	T	A	G	G	G	5e-04	NA	2e-04	0.998
65	A	A	C	A	A	C	T	4e-04	NA	7e-04	0.9985
66	A	A	C	G	A	C	T	4e-04	0	9e-04	0.9989
67	A	C	T	G	A	G	G	4e-04	NA	0	0.9993
68	A	C	T	G	A	C	T	3e-04	0.0038	NA	0.9996
69	A	A	C	G	G	C	G	3e-04	NA	NA	1

70	A	A	C	A	G	T	T	0	NA	3e-04	1
71	A	A	C	A	G	C	G	0	NA	4e-04	1

S6 Table. Association of polymorphisms with the risk of developing COPD.

SNP	Minor Allele	Major Allele	Model	Cases	Controls	χ^2	p-value χ^2	p-value Fisher
rs7041	A	C	Genotypic	30/75/47	81/240/135	0.547	0.761	0.749
			Additive	135/169	402/510	0.011	0.918	0.918
			Allelic	135/169	402/510	0.01	0.920	0.947
			Dominant	105/47	321/135	0.094	0.759	0.759
			Recessive	30/122	81/375	0.298	0.585	0.628
rs10741657	A	G	Genotypic	28/73/51	64/189/203	5.875	0.053	0.049
			Additive	129/175	317/595	5.393	0.021	0.020
			Allelic	129/175	317/595	5.783	0.016	0.019
			Dominant	101/51	253/203	5.635	0.018	0.018
			Recessive	28/124	64/392	1.708	0.191	0.193
rs731236	C	T	Genotypic	31/68/53	74/222/160	1.52	0.468	0.459
			Additive	130/174	370/542	0.446	0.504	0.504
			Allelic	130/174	370/542	0.453	0.501	0.502
			Dominant	99/53	296/160	0.002	0.961	1
			Recessive	31/121	74/382	1.385	0.239	0.265
rs7975232	C	A	Genotypic	36/68/48	100/228/128	1.29	0.524	0.51
			Additive	140/164	428/484	0.069	0.792	0.793
			Allelic	140/164	428/484	0.071	0.791	0.842
			Dominant	104/48	328/128	0.682	0.409	0.411
			Recessive	36/116	100/356	0.202	0.653	0.654
rs1544410	A	G	Genotypic	33/68/51	95/213/148	0.179	0.914	0.925
			Additive	134/170	403/509	0.001	0.974	0.974
			Allelic	134/170	403/509	0.001	0.973	1
			Dominant	101/51	308/148	0.062	0.803	0.84
			Recessive	33/119	95/361	0.053	0.818	0.819
rs2228570	T	C	Genotypic	25/56/71	49/195/212	4.014	0.134	0.137
			Additive	106/198	293/619	0.731	0.393	0.393
			Allelic	106/198	293/619	0.777	0.378	0.398
			Dominant	81/71	244/212	0.002	0.963	1
			Recessive	25/127	49/407	3.467	0.063	0.084
rs11568820	A	G	Genotypic	9/48/95	25/179/252	2.884	0.236	0.233
			Additive	66/238	229/683	1.457	0.227	0.227
			Allelic	66/238	229/683	1.434	0.231	0.247
			Dominant	57/95	204/252	2.437	0.119	0.130
			Recessive	9/143	25/431	0.041	0.838	0.839
rs4646536	G	A	Genotypic	13/50/89	84/176/196	13.85	0.0009	0.0008
			Additive	76/228	344/568	13.85	0.0002	0.0002
			Allelic	76/228	344/568	16.31	0.054	0.0001
			Dominant	63/89	260/196	11.1	0.0009	0.001
			Recessive	13/139	84/372	8.28	0.004	0.003
rs3782130	C	G	Genotypic	26/49/77	63/183/210	3.217	0.200	0.189
			Additive	101/203	309/603	0.038	0.844	0.844
			Allelic	101/203	309/603	0.044	0.833	0.889
			Dominant	75/77	246/210	0.970	0.324	0.348

			Recessive	26/126	63/393	0.987	0.320	0.353
rs10877012	T	G	Genotypic	6/51/95	28/178/250	3.074	0.215	0.232
			Additive	63/241	234/678	3.069	0.079	0.079
			Allelic	63/241	234/678	3.007	0.083	0.09
			Dominant	57/95	206/250	2.736	0.098	0.108
			Recessive	6/146	28/428	1.038	0.308	0.415
rs703842	G	A	Genotypic	7/46/99	32/180/244	6.366	0.042	0.040
			Additive	60/244	244/668	5.936	0.015	0.014
			Allelic	60/244	244/668	5.988	0.014	0.014
			Dominant	53/99	212/244	6.263	0.012	0.014
			Recessive	7/145	32/424	1.105	0.293	0.343
rs4809957	G	A	Genotypic	12/47/93	21/166/269	3.343	0.188	0.189
			Additive	71/233	208/704	0.038	0.845	0.844
			Allelic	71/233	208/704	0.038	0.844	0.874
			Dominant	59/93	187/269	0.228	0.633	0.702
			Recessive	12/140	21/435	2.403	0.121	0.146
rs6068816	T	C	Genotypic	5/28/119	21/99/336	1.375	0.503	0.572
			Additive	38/266	141/771	1.363	0.24	0.243
			Allelic	38/266	141/771	1.592	0.207	0.225
			Dominant	33/119	120/336	1.284	0.257	0.282
			Recessive	5/147	21/435	0.482	0.487	0.644
Chr: cromosoma; NA: not aplicable								
